# A novel gene from the acidophilic bacterium *Leptospirillum* sp. CF-1 and its role in oxidative stress and chromate tolerance

**DOI:** 10.1186/s40659-022-00388-0

**Published:** 2022-05-07

**Authors:** Rivera-Araya Javier, Riveros Matías, Ferrer Alonso, Chávez Renato, Levicán Gloria

**Affiliations:** 1 Biology Department, Faculty of Chemistry and Biology, University of Santiago of Chile (USACH), Santiago, Chile; 2 Núcleo de Química y Bioquímica, Facultad de Ciencias, Universidad Mayor, Santiago, Chile

**Keywords:** Hypothetical proteins, Chromate, Hydrogen peroxide, Oxidative stress, *Och* operon, *Leptospirillum*

## Abstract

**Background:**

Acidophilic microorganisms like *Leptospirillum* sp. CF-1 thrive in environments with extremely low pH and high concentrations of dissolved heavy metals that can induce the generation of reactive oxygen species (ROS). Several hypothetical genes and proteins from *Leptospirillum* sp. CF-1 are known to be up-regulated under oxidative stress conditions.

**Results:**

In the present work, the function of hypothetical gene ABH19_09590 from *Leptospirillum* sp. CF-1 was studied. Heterologous expression of this gene in *Escherichia coli* led to an increase in the ability to grow under oxidant conditions with 5 mM K_2_CrO_4_ or 5 mM H_2_O_2_. Similarly, a significant reduction in ROS production in *E. coli* transformed with a plasmid carrying ABH19_09590 was observed after exposure to these oxidative stress elicitors for 30 min, compared to a strain complemented with the empty vector. A co-transcriptional study using RT-PCR showed that ABH19_09590 is contained in an operon, here named the “*och*” operon, that also contains ABH19_09585, ABH19_09595 and ABH19_09600 genes. The expression of the *och* operon was significantly up-regulated in *Leptospirillum* sp. CF-1 exposed to 5 mM K_2_CrO_4_ for 15 and 30 min. Genes of this operon potentially encode a NADH:ubiquinone oxidoreductase, a CXXC motif-containing protein likely involved in thiol/disulfide exchange, a hypothetical protein, and a di-hydroxy-acid dehydratase. A comparative genomic analysis revealed that the *och* operon is a characteristic genetic determinant of the *Leptospirillum* genus that is not present in other acidophiles.

**Conclusions:**

Altogether, these results suggest that the *och* operon plays a protective role against chromate and hydrogen peroxide and is an important mechanism required to face polyextremophilic conditions in acid environments.

## Background

*Leptospirillum* spp. and other acidophiles face environmental conditions that favor the generation of reactive oxygen species (ROS) [1–4]. Consequently, they may experience oxidative stress, a very deleterious condition where all major cellular components, such as DNA, RNA, proteins, lipids and cofactors can suffer oxidative damage with substantial negative impacts on cellular physiology and activity. *Leptospirillum* sp. CF-1 is an aerobic acidophilic iron-oxidizing bacterium with the capacity to survive in extremely acidic natural or man-made environments, and in high concentrations of dissolved metal(loids) such as Cu, Zn, Al, Cd, Cu, Ni, Cr and As [3, 5]. We have investigated the molecular mechanisms that mediate the antioxidant response in *Leptospirillum* spp., and have established that members of this genus have a highly abundant and efficient thiol/disulfide system [6]. *Leptospirillum* spp. also possesses antioxidant enzymes, like Dyp-type peroxidases (DyP) [7] and cytochrome *c* peroxidases (CcP) [8], which are responsible for eliminating the peroxides generated in the cytoplasm and periplasm, respectively. It has also been established that *Leptospirillum* sp. CF-1 uses a mechanism based on cobalamin (vitamin B_12_) to protect against oxidative conditions and avoid the induction of oxidative stress [3].

The genome of *Leptospirillum* sp. CF-1 has been completely sequenced [9], facilitating genomic, transcriptomic and proteomic studies to provide a better understanding of the adaptive mechanisms to extreme environments. Since no genetic systems have been developed for this microorganism to date, omics approaches have become a very important tool in microbial physiology studies. However, genomic data derived from *Leptospirillum* sp. CF-1 has revealed the existence of a very high number of genes with unknown function (1519 out of 2736; 56%), most of which are present in genetic contexts that are also unknown in terms of function [9]. These hypothetical genes have the regulatory elements required to be transcribed and translated, but the predicted products have no known function or structure, making their biological role and contribution towards adaptation to extreme life impossible to deduce [10]. In the case of strain CF-1, and other strains of the genus, more than half of the annotated genes in its genome still need to be functionally characterized. This proportion is similar to less-characterized microorganisms or extremophiles (> 50%) [11], and substantially higher than in model organisms (25–40%; [12]. For example, in cyanobacterial genomes, 30–60% of the putative proteins are encoded by hypothetical genes depending on the species [10]. A similar profile has been described for extremophilic microorganisms; for example, 36% are hypothetical proteins in *Acidithiobacillus ferrooxidans* and *At. thiooxidans* [13, 14], and 47% of protein-coding genes from *Leptospirillum ferriphilum* are of unknown function [15], a figure that rises to 63% in the case of *Ferrovum myxofaciens* [16]. Although many hypothetical genes may correspond to non-functional entities called pseudogenes [17], it is also likely that many have functions not yet characterized. As such, they could possess novel biological roles that could allow us to gain a deeper and more comprehensive understanding of the mechanisms involved in adaptation to environmental challenges. Interestingly, transcriptomic and proteomic assays carried out in *Leptospirillum* sp. CF-1 have shown a large number of hypothetical genes or proteins that were down or up-regulated under oxidative stress conditions (unpublished observations), suggesting a role of these in the adaptation of *Leptospirillum* spp. to highly oxidative and metal loaded environments.

Bioinformatic studies to characterize hypothetical genes and proteins have traditionally included analyses that search for orthologous genes or sequence similarity to previously characterized proteins, the presence of conserved domains or motifs, the prediction of protein cellular location, and the identification of neighboring genes, among other predictions [18–20]. The utilization of in silico approaches to predict the function of hypothetical genes and proteins has been successfully used in several models of bacterial pathogens [21–23]. However, since most of these analyses are performed based on comparisons with previously-known protein sequences, structures or domains, it becomes evident that there is insufficient data to obtain novel information about the nature, origin and role of predicted hypothetical proteins in less-characterized or extremophilic microorganisms. Thus, in the present work, we have addressed the study of the hypothetical gene ABH19_09590. This gene was heterologously expressed in *Escherichia coli* to evaluate its ability to confer protection against oxidative stress. Furthermore, neighboring genes from *Leptospirillum* sp. CF-1 were studied to assess the genetic context of ABH19_09590. The expression profiles of neighboring genes found in this operon were determined to understand their potential role in chromate tolerance.

## Results

### Selection of hypothetical genes that are upregulated under oxidative stress conditions

A previous study carried out in *Leptospirillum* sp. CF-1 showed that oxidative stress induced with ferric ions (260 mM Fe_2_(SO_4_)_3_, Fe^3+^) for 1 h led to the up-regulation of genes and proteins involved in the synthesis of iron-sulfur centers, protein folding, energy metabolism, synthesis of exopolysaccharides, and metabolism of methionine (data not shown). Interestingly, these experiments also showed changes at the expression level of a number of hypothetical genes and proteins. According to our predictions, 38% of the genes or proteins that were significantly down- or up-regulated corresponded to hypothetical proteins, suggesting that they could be involved in the oxidative stress response of this microorganism and indicating that they could play a pivotal role under environmental stress conditions.

Among all *Leptospirillum* sp. CF-1 genes that showed statistically reliable regulation of expression levels in transcriptomic assays under ferric ion stress, we chose those whose products were originally annotated as hypothetical proteins and were conserved among other members of the *Leptospirillum* genus. In this way, we selected four up-regulated genes: ABH19_09590 (4.7 fold-change), ABH19_06740 (3.6 fold-change), ABH19_07700 (2.8 fold-change), and ABH19_010550 (1.8 fold-change). These genes were evaluated by means of heterologous complementation experiments (described below), to confer protection to *E. coli* against potassium chromate (data not shown). In this initial screen, the ABH19_09590 gene showed a high protective effect, and was thus selected for further analysis and evaluation.

### *E. coli* transformed with ABH19_09590 showed tolerance to heavy metals and oxidative stress conditions

**Fig. 1:**
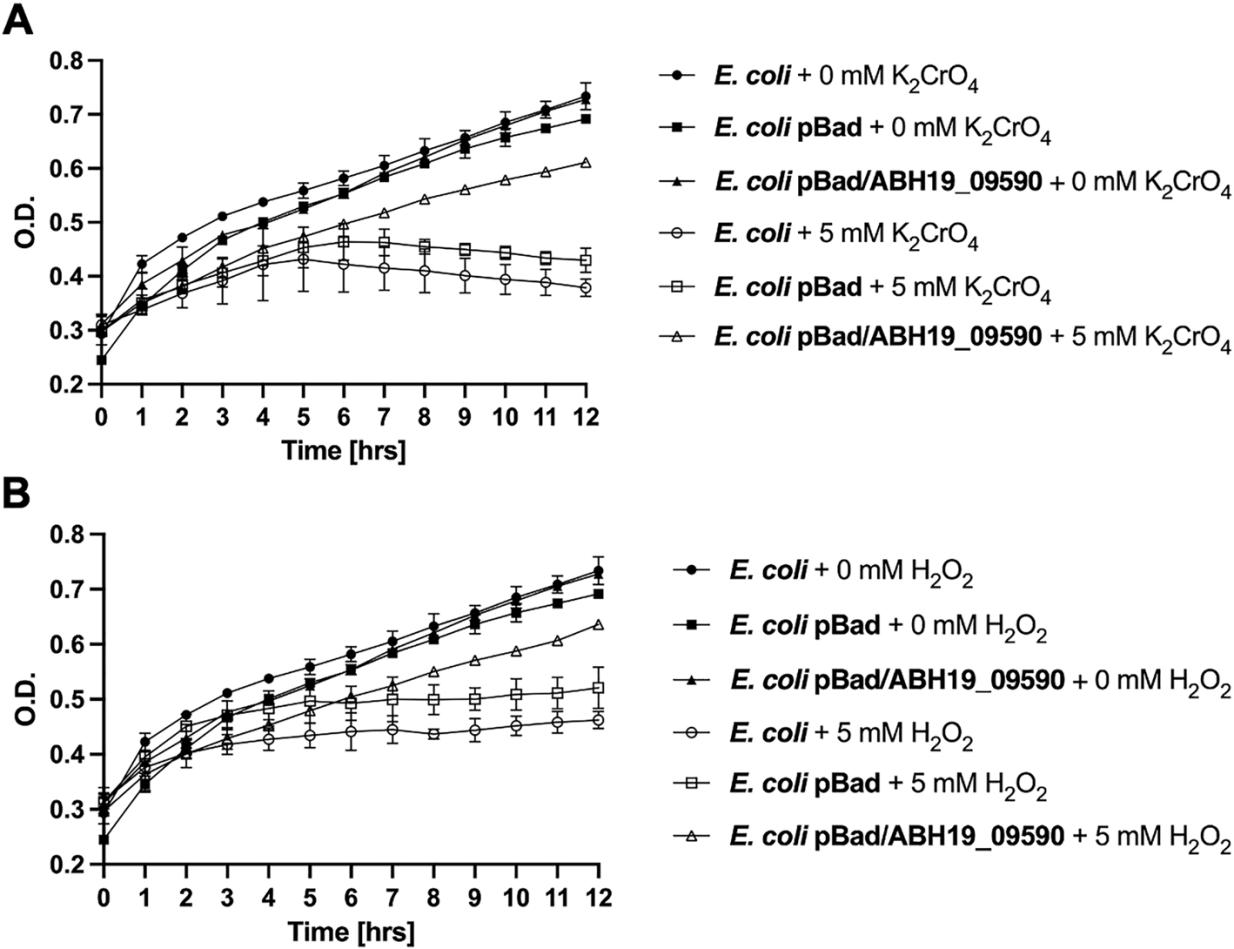
Growth of *E. coli* transformed with ABH19_09590 from *Leptospirillum* sp. CF-1. Cells transformed with plasmid pBADTopo/ABH19_09590 were exposed to **A** 5 mM K_2_CrO_4_, or **B** 5 mM H_2_O_2_. Cell growth was measured each hour at 600 nm absorbance

Since no molecular tools exist for *Leptospirillum* spp., in order to evaluate the functionality of ABH19_09590 gene from strain CF-1, we measured its ability to confer protection against oxidative stress conditions when heterologously-expressed in *E. coli*. The selected gene was cloned into the arabinose-inducible expression vector pBATopo TA, as described in Methods, and used to transform *E. coli* (Top 10 strain). The transformed cell culture was grown until OD_600_ 0.5 and expression of the cloned gene was induced with L-arabinose for 1.5 h. Subsequently, the cultures were exposed to oxidative stress culture conditions as described in Methods. The ferric iron initially used to induce oxidative stress in *Leptospirillum* spp. at acidic pH [3], has a high tendency to precipitate and form aggregates with organic components of the circumneutral medium for *E. coli* cultivation therefore potentially affecting the quantification of its biological effect in this bacterium. For this reason in this study we chose potassium chromate and hydrogen peroxide to induce oxidative stress. The effect of these as oxidative stress elicitors in *Leptospirillum* spp. has also been previously reported [3, 24]. In agreement with those previous studies, the exposure of wild type *E. coli* to 5 mM K_2_CrO_4_ or 5 mM H_2_O_2_ led to a significant decrease in cell growth (K_2_CrO_4_: 50%, and H_2_O_2_: 63%) at 12 h, as compared to cells that were not exposed to these oxidant agents (100%; Fig. 1). Interestingly, as shown in Fig. 1A and B, heterologous ABH19_09590 transformation exerted a significant positive effect in restoring cell growth in the presence of 5 mM K_2_CrO_4_ (81% at 12 h) and 5 mM H_2_O_2_ (87% at 12 h). In this way, these data suggest that ABH19_09590 could be exerting a protective role against the oxidative effect of chromate and ROS.

### ABH19_09590 attenuates ROS generation in *E. coli*

**Fig. 2:**
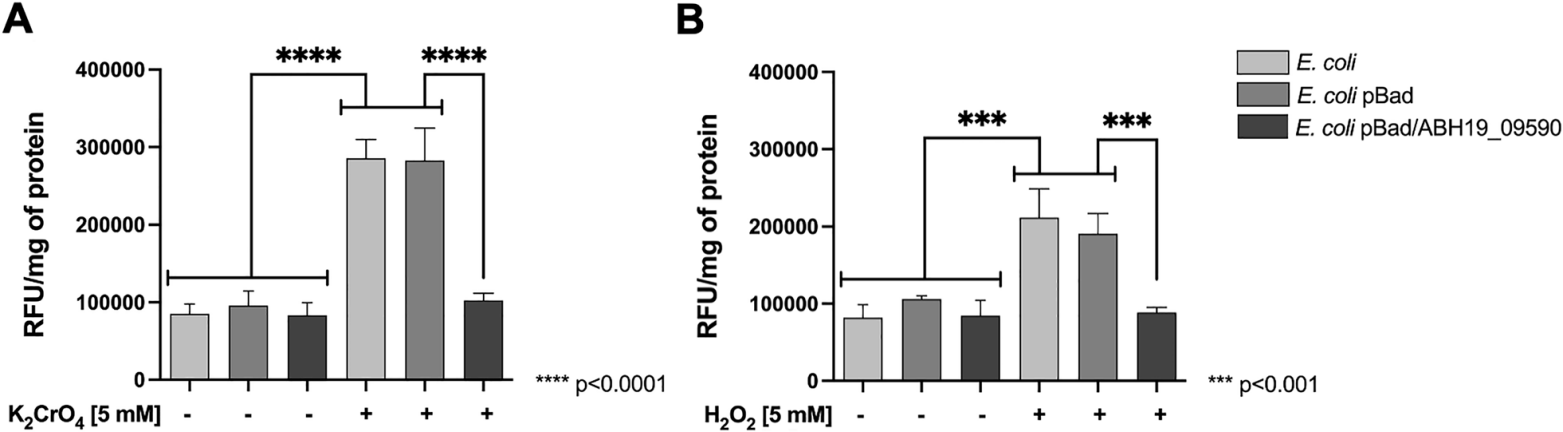
ROS levels of *E. coli* transformed with ABH19_09590 from *Leptospirillum* sp. CF-1. Cells were stressed with **A** 5 mM K_2_CrO_4_, or **B** 5 mM H_2_O_2_ for 30 min. Cytoplasmic ROS content is expressed as relative fluorescence units (RFU) of the activated fluorescent probe H_2_DCFDA per mg of protein

To determine whether the ABH19_09590 gene product has a suppressive effect on ROS generation, the intracellular ROS levels in *E. coli* exposed to oxidant agents were measured. As shown in Fig. 2, empty vector cells exposed to 5 mM K_2_CrO_4_ or 5 mM H_2_O_2_ for 30 min showed significantly increased ROS contents (by 335% and 258%, respectively) as compared to control cells that were not exposed to stress (100%). Interestingly, and consistent with results observed in cell growth, exposure of transformed and induced cells to 5 mM K_2_CrO_4_ or 5 mM H_2_O_2_ for 30 min, led to a much more minor increase in ROS content (by 120% and 108%, respectively) compared to control cells without exposure (100%). It should be noted that a decrease in intracellular ROS level was not observed in stressed cells harboring the empty pBAD vector. These results suggest that the protein encoded by ABH19_09590 alleviates the cytotoxic effect of oxidative stress elicitors by directly or indirectly scavenging ROS and restoring the redox balance of the cell.

### Analysis of the genetic context of the ABH19_09590 gene

**Fig. 3:**
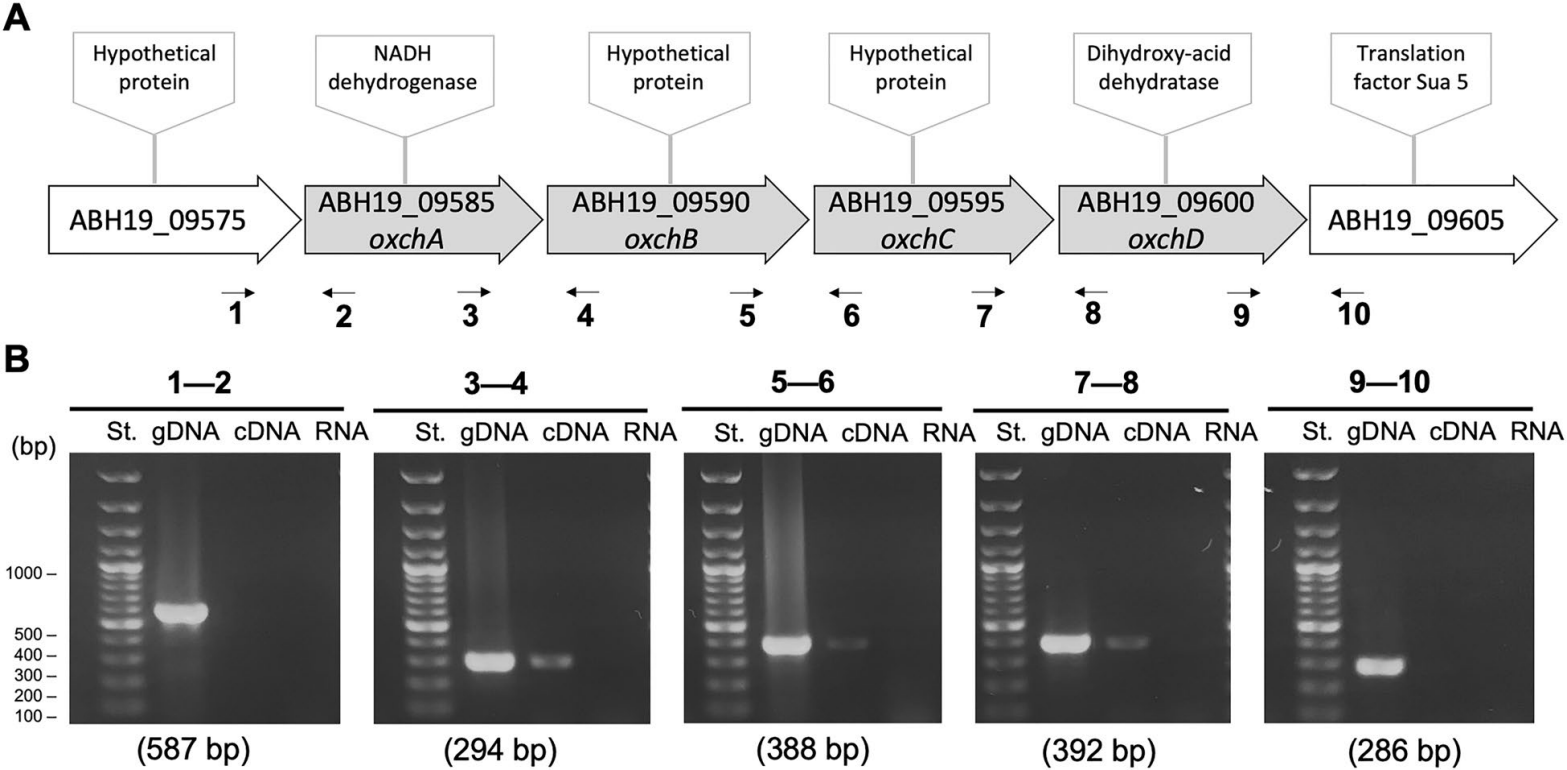
Genetic organization of the *och* operon of *Leptospirillum* sp. CF-1. **A** Schematic map of the *och* operon. Primers designed in each intergenic region are indicated below the operon. **B** RT-PCR amplification of intergenic regions. The negative control corresponds to RT-PCR amplification without reverse transcriptase, whilst the positive control corresponds to standard PCR amplification with genomic DNA. Primer pairs used for amplification are indicated above each panel and the amplification product sizes are indicated below each panel

In a previous study, we generated a high-quality sequence of the whole genome of strain CF-1 [9]. For this reason, and in order to obtain additional information on the potential role of the ABH19_09590 gene and its encoded protein, we analyzed its genetic context. Immediately upstream from ABH19_09590, genes that encode for a hypothetical protein (*ABH19_09575*) and for putative NADH dehydrogenase subunit L (NuoL, *ABH19_09585*) are found, while downstream, genes encoding a hypothetical protein (ABH19_09595), a possible dihydroxy-acid dehydratase (DHAD, ABH19_09600) and the translational factor Sua5 (ABH19_09605) were detected. The six candidate genes were analyzed to evaluate co-transcription using RT-PCR, in order to define whether ABH19_09590 is contained in an operon. RT-PCR experiments clearly show that ABH19_09585, ABH19_09590, ABH19_09595 and ABH19_09600 are co-transcribed (Fig. 3), demonstrating that they are expressed and form part of an operon that we have called the “*och* operon” (oxidative stress and chromate response). The physical genetic arrangement of the o*ch* operon, including the flanking non-co-transcribed genes (ABH19_09575 and ABH19_09605) is shown in Fig. 3. The co-transcription of these four genes suggests that they are functionally related and similarly activated in response to physiological signals. Therefore, these results strongly suggest the involvement of the four genes of the *och* operon in the oxidative stress response of *Leptospirillum* spp.

### The *och* operon is up-regulated in *Leptospirillum* sp. CF-1 under oxidative stress

**Fig. 4:**
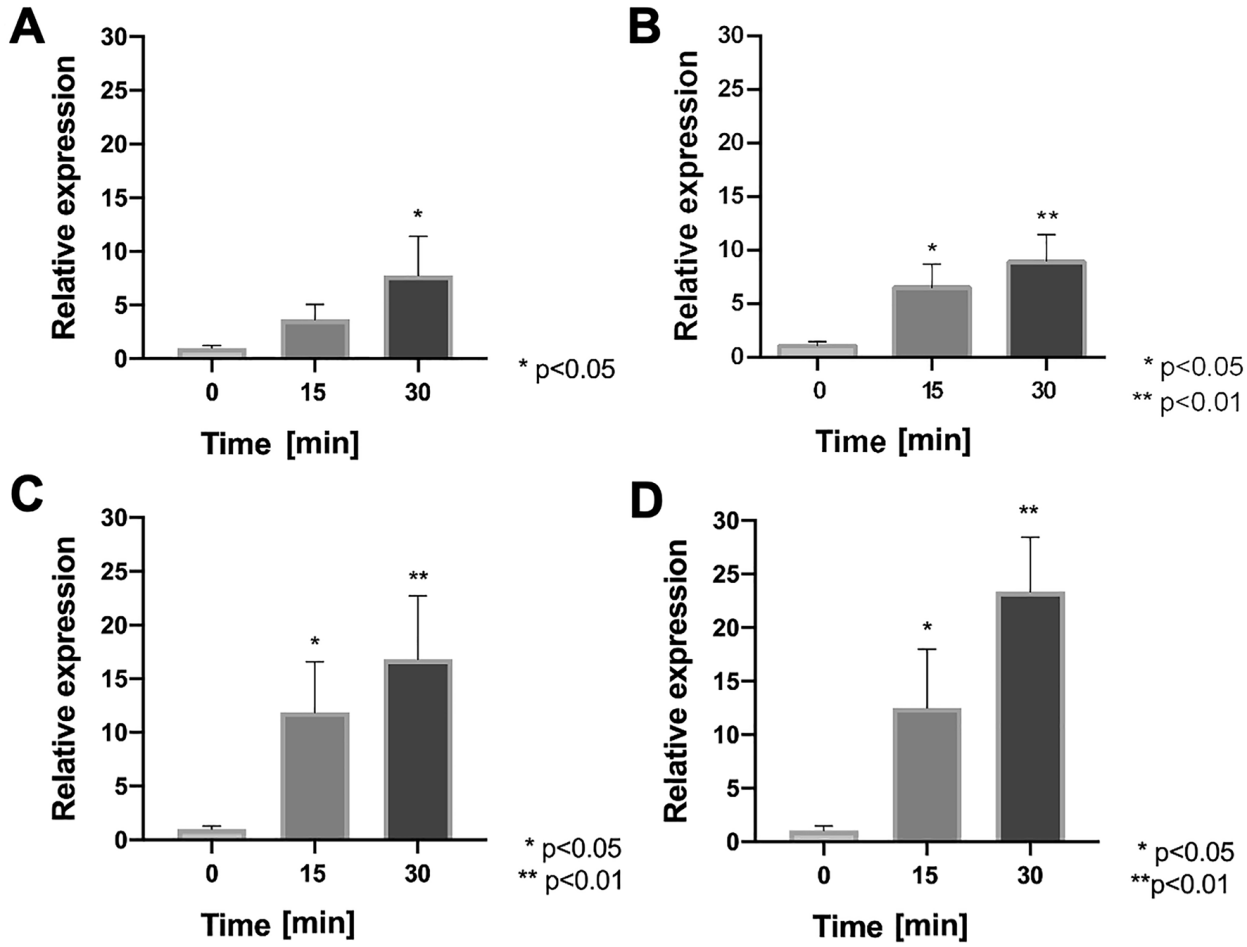
Effect of chromate exposure on the relative mRNA levels of the *och* operon of *Leptospirillum* sp. CF-1. The relative expression of **A** ABH19_09585, **B** ABH19_09590, **C** ABH19_09595 and **D** ABH19_9600 encoding genes was evaluated in cells treated with 5 mM K_2_CrO_4_ for 15 and 30 min. Data represent the average of three independent experiments (bars indicate the value range). Statistical analysis was carried out by the ANOVA Test

To evaluate the role of the *och* operon in oxidative defense, the mRNA levels of each of the four genes contained in this operon were evaluated using RT-qPCR assays when *Leptospirillum* sp. CF-1 was exposed to oxidative stress conditions. Unfortunately, due to the high reactivity between the ferrous ion and hydrogen peroxide, and the subsequent generation of radicals that promote oxidative damage to biomolecules, exposure of iron-oxidizing microorganisms to peroxide usually leads to difficulties in purification of RNA with a quality suitable for performing RT-qPCR experiments. For this reason, oxidative stress induction in *Leptospirillum* sp. CF-1 was assayed with 5 mM K_2_CrO_4_ for 15 and 30 min, without the addition of H_2_O_2_. As can be observed in Fig. 4, the transcript levels of the four genes of the *och* operon increased significantly by 2.8–12.5 fold after 15 min, and by 7.8–23.5 fold after 30 min in cells exposed to potassium chromate. These data strongly suggest a relevant role of the *och* operon in the physiological oxidative stress response of *Leptospirillum* sp. CF-1.

### In-silico predictions of the proteins encoded by the *och* operon from *Leptospirillum* sp. CF-1

To characterize the gene products that make up the *och* operon, an *in-silico* analysis was performed:

***ABH10_09585* (*ochA*)** The protein encoded by the first gene of the operon from strain CF-1 is predicted to contain 553 amino acids with a theoretical molecular mass of 59.6 kDa and a pI of 9.44. The protein is similar to the membrane-bound subunits L (NuoL) of NADH:ubiquinone oxidoreductase (54% similarity to NuoL subunit from *Shewanella* sp.). NuoL has a role in proton pumping in complex I of the respiratory chain. However, in many bacterial species this protein is a stand-alone Na^+^/H^+^ antiporter not coupled with oxidoreduction, but displaying a more general role in multiple resistance mechanisms and pH adaptation [25, 26].

***ABH10_09590* (*ochB*)** The analysis of the deduced amino acid sequence of ABH19_09590 of *Leptospirillum* sp. CF-1 indicates that the predicted encoded protein possesses 823 amino acids with a theoretical molecular mass of 92 kDa. The prediction shows that this protein harbors five transmembrane domains and lacks domains of known function. However, a DUF2309 domain of unknown function is predicted. DUF2309 is a non-characterized, but widely distributed and conserved domain, spanning 748.4 amino acids on average [27]. Interestingly, this predicted DUF from the deduced ABH19_09590 protein possesses a CXXC active-site motif between amino acids 530 and 533. This motif is highly conserved in most DUF2309 domains and is similar to that reported in iron-sulfur proteins, thioredoxins and oxidoreductases [28]. We postulate that the presence of the CXXC motif may contribute to the formation, isomerization, or reduction of disulfide bonds, or determine other redox functions in the ABH10_09590 protein that could explain the in vivo effect on the growth and ROS concentration under oxidative conditions, when expressed in *E. coli* (Figs. 1 and 2).

***ABH10_09595* (*ochC*)** The encoded hypothetical protein is predicted to have 823 amino acids, a mass of 92 kDa and to be located at the cytoplasmic membrane. It does not possess conserved domains or motifs that could give insights into its role in strain CF-1.

***ABH10_09600* (*ochD*)** The protein encoded by the fourth gene of the operon corresponds to a putative dihydroxy-acid dehydratase (DHAD), with 558 amino acids and 59.2 kDa. This protein participates in the metabolism of amino acids, and plays a key role in the biosynthesis of valine, leucine and isoleucine from Archaea, Bacteria and Eukarya [29]. Also, it has been shown to be involved in the biosynthesis of D-pantothenate and coenzyme A [30]. More recently, it has been described that up-regulation of the DHAD protein in *Staphylococcus* sp. #NIOSBK35 facilitates its survival and growth at high concentrations of Cr(VI), conferring the capability to deal with this strong oxidizing agent [31]. It has been proposed that the upregulation of DHAD could be indicative of enhanced cellular activity as a strategy to cope with the stress induced by Cr(VI). According to our inspection, dihydroxy-acid dehydratase from *Leptospirillum* sp. CF-1 is a predicted cytoplasmic protein with high similarity to DHAD from *E. coli* (45% identity and 63% similarity).

Interestingly, genes that encode ABH19_09585, ABH19_09590, ABH19_09595, ABH19_09600 proteins are detected in the genomes of a number of other acidophilic microorganisms including *Leptospirillum ferriphilum*, *Acidiferrobacter thiooxydans*, *Acidibacillus sulfuroxidans*, *Acidithiobacillus ferrivorans*, *Acidithiobacillus ferrooxidans*, *Sulfobacillus acidiphilus*, *Ferrovum* sp., *Ferrithrix thermotolerans*, *Sulfobacillus thermosulfidooxidans*, and *Acidihalobacter prosperus*, among others, with an identity and query cover of over 47% and 74%, respectively (Table 1). However, with the exception of the member from the *Leptospirillum* genus, in these microorganisms the genes are not organized into operons, and appear dispersed throughout the genome.

**Table 1:** Comparison of each gene from the *och* operon of *Leptospirillum* sp. CF-1 with other acidophiles

Microorganism	Identity (%)			
	ABH19_09585	ABH19_09590	ABH19_09595	ABH19_09600
*Leptospirillum ferriphilum*	100	99	80	99
*Acidiferrobacter thiooxydans*	57	61	54	–
*Acidibacillus sulfuroxidans*	57	57	–	–
*Acidithiobacillus ferrivorans*	52	59	54	72
*Acidithiobacillus ferrooxidans*	52	59	54	72
*Sulfobacillus acidophilus*	51	54	–	–
*Ferrovum sp.*	52	54	55	72
*Ferrithrix thermotolerans*	48	56	–	–
*Sulfobacillus thermosulfidooxidans*	47	53	–	–
*Acidihalobacter prosperus*	51	57	59	–
Query cover	74–100%	98–100%	93–99%	99%

## Discussion

Acidophilic bacteria must maintain the intracellular ROS level under control in order to survive the low pH and/or the toxic levels of heavy metals in their environment. This implies that these bacteria must have developed sophisticated mechanisms to reduce the levels of ROS and repair damaged biomolecules. Members of the genus *Leptospirillum* grow under these environmental conditions in the absence of canonical antioxidant systems, namely superoxide dismutase (SOD), catalase (CAT), and glutathione [1, 3]. However, alternative molecular mechanisms to maintain redox homeostasis have been described in *Leptospirillum* spp., including the thiol/disulfide system [6], Dyp-type peroxidases (DyP) [7], cytochrome *c* peroxidases (CcP) [8] and even the use of cobalamin to protect against oxidative conditions [3]. In the present study, we generated experimental evidence that directly or indirectly links the protein encoded by the hypothetic gene ABH19_09590 of *Leptospirillum* sp. CF-1 with protection against oxidative damage. The results showed that *E. coli* cells overexpressing the gene product of the ABH19_09590 gene were able to grow after exposure to the oxidant metal ion Cr(VI) and the oxidative specie H_2_O_2_, compared to negative controls. In addition, the measurement of the intracellular ROS levels generated by the pro-oxidant agents reinforced the fact that all *E. coli* cultures were exposed to oxidative stress conditions. Interestingly, the culture carrying the plasmid with ABH19_09590 gene had ROS levels similar to those found in control conditions, supporting its antioxidant function.

Additional hints regarding potential biological functions can arise from data concerning the genomic context of hypothetical genes [11]. Thus, we performed a co-transcription analysis to determine whether the ABH19_09590 was part of an operon. The results demonstrated that this gene is co-transcribed with genes encoding NuoL (ABH19_09585), a hypothetical protein (ABH19_09595) and a DHAD (ABH19_09600), altogether conforming the operon denominated “*och*”. Additionally, in line with our previous unpublished observations regarding the upregulation of ABH19_09595 under oxidative stress (with ferric iron), strain CF-1 exposed to chromate exhibited significantly enhanced mRNA levels of each gene from this cluster, suggesting the up-regulation of the *och* operon under the oxidant conditions encountered in these extreme acidic environments. The *in-silico* analysis of the amino acid sequence of the ABH19_09590 gene revealed the presence of a CXXC active site motif, as found in thiol-disulfide oxidoreductases belonging to the thioredoxin superfamily. Their two cysteines usually exist in an oxidized (disulfide bonded) or reduced (unpaired) state, which allow disulfide bonds to generate changes in the redox state and/or conformation of their target proteins [28, 32], which could explain the protective effects observed. The neighboring gene encodes a putative membrane localized NuoL. As a potential proton pump, in the absence of other proteins belonging to complex I of the respiratory chain, NuoL could be involved in pH adaptation [25, 26]. On the other hand, the DHAD protein could confer the ability of the bacterium to deal with Cr(VI) by enhancing cellular activity [31]. Therefore, this in silico evidence strongly suggests that the *och* operon could be a response mechanism against oxidative stress, and more specifically to chromate in *Leptospirillum* sp. CF-1.

Comparative genomics has shown that a substantial fraction of the genes in sequenced genomes encode conserved hypothetical proteins that are found in organisms from several phylogenetic lineages and have no known function [11]. Based on a bioinformatic search within the genomes of different acidophilic iron-oxidizing microorganisms, the genes from the *och* operon are apparently conserved, which could suggest their importance for subsisting in extreme bioleaching environmental conditions. However, it is important to highlight that only bacteria from the *Leptospirillum* genus possess an operon-like organization. Therefore, whether the full operon confers better fitness under oxidative conditions to *E. coli*, compared to each gene alone, is a task that should be addressed.

In bacteria, Cr(VI) is imported via anion transporters, and sulfate importers especially seem to represent a major import pathway due to the structural similarity of these two oxyanions [33, 34]. Inside the cell, Cr(VI) generates active intermediates Cr(V) and/or Cr(IV), free radicals, and Cr(III) as the final product. Cr(III) affects DNA replication, causes mutagenesis, and alters the structure and activity of enzymes, reacting with their carboxyl and thiol groups [34–37]. Therefore, ROS are generated during the reduction of Cr(VI) to Cr(III). However, several bacterial mechanisms of Cr(VI) detoxification have been described in detail, such as the chromium resistance (*chr*) operon [33, 38], efflux pumps [38, 39], extracellular Cr(VI) reduction [40–43], enzymes involved in the detoxifying processes [44–46], and repair of DNA lesions [47–50]. In the case of acidophilic microorganisms, Cr(VI) precipitation has been identified in *At. ferrooxidans,* which can tolerate Cr(III) up to 75 mM during growth on ferrous sulphate [51]. In addition, *Acidiphilum cryptum* JF-5 has the ability to reduce Cr(VI) under aerobic and anaerobic conditions via two mechanisms, a NADPH-dependent chromate reductase and a *c*-type cytochrome (ApcA) [52]. In strain CF-1, no genes related to Cr(VI) resistance were found by bioinformatic searches. However according to the results generated in this work, we propose that the ABH19_09590 gene product could be part of a new chromate resistant mechanism achieved by direct Cr(VI) reduction, or reduction of the ROS generated.

## Conclusions

The ABH19_09590 gene from *Leptospirillum* sp. CF-1 has a role increasing tolerance and attenuating ROS generation when transformed *E. coli* are exposed to chromate and hydrogen peroxide. The ABH19_09590 gene encodes a DUF2309 domain-containing protein likely involved in thiol/disulfide exchange reactions. This gene forms part of the *och* operon that also contains genes encoding a NuoL subunit of NADH:ubiquinone oxidoreductase (ABH19_09585), a hypothetical protein (ABH19_09595) and a dihydroxy-acid dehydratase (ABH19_09600). The genes from the *och* operon are up-regulated in strain CF-1 under chromate exposure. Finally, the *och* operon is a unique genetic determinant from members of the *Leptospirillum* genus and may represent an important mechanism needed to face polyextremophilic conditions of acid environments. Additional efforts are required to elucidate the role of hypothetical genes in extremophilic microorganisms.

## Methods

### Bacterial strains and growth conditions 

*Leptospirillum* sp. CF-1 was grown as described previously by Ferrer et al. [3]. Briefly, it was grown in 9 K BR medium containing 18.4 g/L ferrous sulfate (Fe_2_SO_4_·7H_2_O) at 37 °C with constant stirring at 180 rpm. *E. coli* Top 10 was grown in Luria–Bertani (LB) under aerobic conditions at 37 °C and constant stirring at 180 rpm. The growth of all recombinant *E. coli* strains was estimated by measuring optical density (OD) at 600 nm.

### DNA isolation

*Leptospirillum* sp. CF-1 was grown until the late exponential phase. Cells were harvested by centrifugation at 8000 ×*g* for 15 min and washed once with acid water (10 mM H_2_SO_4_) and twice with 10 mM sodium citrate pH 7.0. Genomic DNA 1 was isolated using the Wizard® Genomic DNA Purification Kit (Promega) according to the manufacturer’s instructions and stored at − 20 °C until further use.

### Cloning of ABH19_09590 gene in an expression vector

The ABH19_09590 gene was cloned in the pBADTopo TA expression vector (Thermo Fisher Scientific, Walthman, MA, USA) to generate pBADTopo/ABH19_09590. The cloning was performed using the PCR product obtained from ABH19_09590 gene with genomic DNA from *Leptospirillum* sp. CF-1 as a template and specific primers (Table 2). The ligation product was obtained according to the manufacturer’s instructions, and then introduced into *E. coli* Top 10 using the CaCl_2_ transformation protocol [53]. To select transformants, the transformation mix was plated on LB-Agar media containing 100 μg/mL ampicillin.

**Table 2:** Primers used in this study

	Gene	Sequence	Tm	Amplicon (bp)
Cloning	ABH19_09590	(F)ATGAACAACACTGCCGGACAC	61	2469
		(R)CTTTCCGTCCGGGCACATT		
RT-PCR	ABH19_09575	(F)GCGATGACAGGAAGAACCAT	61	587
	ABH19_09585	(R)AATAAGTGGCGGGTTTGGAC		
	ABH19_09585	(F)CTGGTTCAGCAGTTTCTTTCG	60	294
	ABH19_09590	(R)GATTGACGGCCACAAAATTC		
	ABH19_09590	(F)ACCTCATCGAAAACGAATGG	61	388
	ABH19_09595	(R)GATCACCGCCTCTTTTCCAT		
	ABH19_09595	(F)GCTCGTTGTGTCCCGTCT	60	392
	ABH19_09600	(R)ACAATCGGCTTGCCAAAAT		
	ABH19_09600	(F)GTGCCGGAGGAGGAATTT	60	286
	ABH19_09605	(R)CAGACCGTAGACCGTTTCG		
RT-qPCR	ABH19_09585	(F)ATCATCTACACGGGAGCCTTT	60	110
	ABH19_09585	(R)GTCAGGGCAATGGAAAGAAGT		
	ABH19_09590	(F)ACAACTTCTGGAACGGATGG	60	105
	ABH19_09590	(R)GCCTCTTCGAGATCGTCTTTT		
	ABH19_09595	(F)ACCTGGAGTGTCTGGTCTGG	60	111
	ABH19_09595	(R)TGACCTCCTCCCAGTCTTTC		
	ABH19_09600	(F)GTCCCTGTTCTGTGCGATCT	60	104
	ABH19_09600	(R)CCGTTCGACAACAGGATTTT		

*(F)* Forward, *(R)* Reverse

### Heterologous expression assay

Wild-type *E. coli* Top 10, or cells carrying pBadTopo/ABH19_09590 or pBadTopo alone (empty vector) were grown until OD_600_ = 0.2–0.3. Gene expression was induced with 0.2% L-arabinose during 1.5 h. Afterwards, cultures were diluted (OD_600_ = 0.2–0.3) with fresh medium containing 0.2% L-arabinose and treated with 5 mM K_2_CrO_4_, or 5 mM H_2_O_2_. Controls with non-exposed cells were included. Bacterial growth was monitored hourly by spectrophotometry at OD_600_ for 12 h.

### Determination of ROS Levels

The oxidant-sensitive probe H_2_DCFDA (2′,7′-dichlorodihydrofluorescein diacetate) [54] was used to determine the intracellular level of total ROS according to [3]. For this purpose, 15 mL of wild-type *E. coli* Top 10, or cells harboring pBadTopo/ABH19_09590, or pBadTopo alone were cultured overnight in LB media containing 100 μg/mL ampicillin. Afterwards, cultures were centrifuged and resuspended in fresh medium with 100 μg/mL ampicillin until OD = 0.5. Subsequently, cell cultures were induced with 0.2% L-arabinose during 1.5 h, and further exposed to oxidative stress with 5 mM K_2_CrO_4_ or 5 mM H_2_O_2_ for 30 min. Controls with non-exposed cells were included. After stress induction, cells were collected by centrifugation, disrupted by sonication and treated with H_2_DCFDA fluorescent probe as described in [3]. The emitted fluorescence values were normalized to the respective protein concentration that was determined as described by [55].

### RNA isolation and cDNA synthesis

*Leptospirillum* sp. CF-1 was grown until the late exponential phase. Cells were harvested by centrifugation at 8000 ×*g* for 15 min and washed once with acid water (10 mM H_2_SO_4_) and twice with 10 mM sodium citrate pH 7.0. Washed cells were suspended in 9 K BR medium (without Fe_2_SO_4_) and incubated with 5 mM K_2_CrO_4_ for 15 or 30 min, or 5 mM H_2_O_2_ for 30 min. Cells were collected by centrifugation at 8000 ×*g* for 10 min, and washed twice with 10 mM sodium citrate pH 7.0. RNA was isolated using the RNeasy Mini Kit (Qiagen). DNA was removed by DNase I treatment (New England, Biolabs) according to the manufacturer’s instructions. cDNA synthesis was carried out with the AffinityScript qPCR cDNA Synthesis kit (Agilent Technologies). The reaction mixture of 20 µl contained First Strand master mix, 0.1 µg/µl random primers, Affinity Script RT/RNase Block enzyme mixture and 1 µg RNA. cDNA synthesis was carried out at 25 °C for 5 min, 42 °C for 15 min and then the enzyme was inactivated at 95 °C for 5 min. cDNA was stored at − 80 °C until further use.

### Determination of gene co-transcription of *och* operon: RT-PCR reaction

Primers for PCR reactions were designed using the available gene sequences of *Leptospirillum* sp.CF-1 [9]. The GoTaq® DNA Polymerase kit (Promega) was used for PCR amplification according to the provider´s instructions. The characteristics of each primer pair and size of amplicons are shown in Table 2.

### Determination of relative levels of RNA: quantitative PCR reaction

Primers of *Leptospirillum* sp. CF-1 designed for qPCR reactions are shown in Table 2. The KAPA SYBR FAST qPCR kit (Kapabiosystems) was used for qPCR amplification according to the manufacturer’s instructions. The qPCR conditions were an initial denaturation at 95 °C for 5 min, followed by 40 cycles of denaturation (95 °C for 30 s), annealing (60 °C for 20 s) and extension (72 °C for 10 s). All reactions were performed in a StepOne Real-Time PCR system (Applied Biosystems). The relative abundance of each gene versus a constitutively expressed gene (16S rRNA gene) was determined by the comparative Ct (∆∆Ct) method.

### In-silico analysis of gene products

Predicted amino acid sequences derived from genes were used to perform a BlastP [56] search of the NCBI non-redundant data base. Only the best hits were accepted as evidence of putative orthologs. Translated proteins were further characterized using bioinformatics tools: primary structure similarity relations were determined using ClustalW 1.8 [57], structural motif predictions were determined using Prosite [58] and peptide domain predictions were determined using ProDom [59]. Theoretical isoelectric points (pI) and molecular weights (MW) were predicted using Compute pI/MW [60], whilst bacterial protein subcellular localization was predicted using PSORTb v3.0 [61].

### Statistical analysis

Statistical analysis was performed using the one-way ANOVA test followed by Tukey’s, using GraphPad Prism 5. The differences were considered to be significant at P < 0.05.

## Data Availability

Not applicable.

## Abbreviations

ROS: Reactive oxygen species
SOD: Superoxide dismutase
CAT: Catalase
DHAD: Dihydroxy-acid dehydratase
DyP: Dyp-type peroxidases
CcP: Cytochrome *c* peroxidase
OD: Optical density
LB: Luria–Bertani
H_2_DCFDA: 2′,7′-Dichlorodihydrofluorescein diacetate
